# Intelligent Algorithm-Based Magnetic Resonance for Evaluating the Effect of Platelet-Rich Plasma in the Treatment of Intractable Pain of Knee Arthritis

**DOI:** 10.1155/2022/9223928

**Published:** 2022-05-26

**Authors:** Bing Huang, Yun Huang, Xin Ma, Yuequn Chen

**Affiliations:** Department of Orthopaedics, Jiangyin Lingang Hospital, Jiangyin 214443, Jiangsu, China

## Abstract

The application of intelligent algorithms in the treatment of intractable pain of patients with platelet-rich plasma (PRP) knee osteoarthritis by magnetic resonance was investigated. The automatic diagnosis of magnetic resonance knee osteoarthritis was established with multiple intelligent algorithms, including gray projection algorithm, adaptive binarization algorithm, and active shape model (ASM). The difference between automatic magnetic resonance detection indexes of the patients with knee osteoarthritis and artificial measurement results was analyzed. The included patients received PRP treatment. Knee osteoarthritis MRI osteoarthritis knee scores (KOA MOAKS) and Western Ontario and McMaster Universities arthritis index (WOMAC) before and after treatment were compared. The results showed that the results of knee osteoarthritis scores, inferior angle of femur, superior angle of tibia, and tibiofemoral angle (TFA) by automatic magnetic resonance diagnostic model were entirely consistent with artificial detection results. After the treatment, the total scores of knee lateral area, interior area, central area, and patellar area were all remarkably lower than those before the treatment (*P* < 0.05). After the treatment, knee KOA MOAKS scores and WOMAC scores were both lower than those before the treatment (*P* < 0.05). Visual analogue scale (VAS) scores 1 week, 2 weeks, and 3 weeks after the treatment were decreased compared with those before the treatment (*P* < 0.05). Relevant studies indicated that intelligent algorithm-based automatic magnetic resonance diagnostic knee osteoarthritis model showed good utilization values, which could provide the reference and basis for the treatment of the patients with knee osteoarthritis.

## 1. Introduction

Osteoarthritis (OA) is a common articular disease, which usually among middle-aged and elderly people. The main clinical symptoms include acute pain and mobility loss. The main pathological features of OA are articular cartilage degeneration, subchondral bone changes, and nonbacterial synovitis [1]. The pathological basis of knee OA (KOA) is cartilage degeneration [2]. The main therapeutic methods for KOA patients include drug therapy, articular cavity injection therapy, and surgical therapy. Surgical therapy is adopted mainly for the patients with stage IV KOA and some patients with stage III KOA. The cost of surgical therapy is high. Total knee arthroplasty (TKA) is a surgical therapeutic method of improving the pains among the patients with KOA at middle and late stages and restoring their functions. KOA can alleviate patients' pains and cure inflammation as quickly as possible. However, 8% to 13% of patients suffer from continuous intractable pain after TKA [3]. There is no effective therapeutic scheme for unknown intractable pain. Substance P (SP) and calcitonin gene-related protein (CGRP) may be the cause of pains after TKA. Relevant studies show that botulinum toxin A (BoNT/A) has anticholinergic and analgesic effects and can relieve patients' pains [4]. In addition, drug therapy also results in the incidence of complications. Long-term use of nonsteroidal anti-inflammatory drugs may cause gastrointestinal reaction and other side effects [5]. Platelet-rich plasma (PRP) is a platelet concentrate from the patient's own blood. According to relevant studies, PRP can effectively improve the clinical symptoms of KOA patients by regeneration repair and elimination of inflammation [6]. Besides, Louis et al. [7] pointed out that PRP showed no obvious therapeutic effects in KOA treatment compared with sodium hyaluronate. The clinical effect of PRP in KOA treatment was still controversial.

At present, the main methods of diagnosing KOA include X-ray image, magnetic resonance imaging (MRI), computed tomography (CT) image, and ultrasonic image. CT with high-density resolution can display the minor changes in articular structure. However, it cannot show synovial membrane and cartilage structures because it has ionizing radiation [8]. Ultrasound is fast, noninvasive, and cheap. It can be used for the diagnosis of OA. Nevertheless, it cannot fully show the minor changes of joints [9]. Plain X-ray can better show skeletons and soft tissues and recognize narrow articular space. Magnetic resonance can measure the thickness and volume of cartilage accurately. Besides, it possesses good application values in the measurement of lesion positions [10]. In recent years, intelligent algorithms are applied in medical image processing and analysis more and more widely. Currently, a large number of intelligent algorithms are applied in MRI image processing, mainly including spectrum-based segmentation methods, region-based segmentation methods, clustering-based segmentation methods, and deep learning-based segmentation methods. Intelligent algorithms show positive application values in the processing and segmentation of MRI images. Traditional machine learning method requires precise feature extraction. The establishment of medical image models requires considerable professional knowledge. Once the feature model is established, it can hardly vary with image data. Its quality has great influences on image segmentation effects. In addition, it shows poor portability with inconvenient application. Deep learning technology has strong feature learning capacity, and it shows more significant advantages with higher application values compared with these machine learning algorithms.

In summary, the clinical effect of PRP in the treatment of KOA is still controversial, and there is no effective magnetic resonance image segmentation method. In this study, the knee region of interest (ROI) was segmented and extracted by a variety of intelligent algorithms to achieve automatic diagnosis of magnetic resonance KOA. Moreover, 23 KOA patients were treated with PRP to evaluate the clinical effect of PRP in magnetic resonance diagnosis of KOA intractable pain and provide a reference for the diagnosis and treatment of KOA patients.

## 2. Materials and Methods

### 2.1. Study Object

Twenty-three patients with KOA admitted to hospital between July 2020 and August 2021 were selected as the study objects. All patients were performed with PRP and MRI detection before and after treatment. There were 14 male patients aged between 43 and 75, and their average age was 53.44 ± 6.15. There were 9 female patients aged between 42 and 73. Their average age was 52.6 ± 5.82. This study had been approved by Ethics Committee of Hospital, and the included patients had signed informed consent forms.

  Inclusion criteria: patients performed with image detection and the assessment of degenerative change level of knee joint before and after treatment; patients conforming to KOA diagnostic standards revised by American College of Rheumatology [11]; patients with pain history for over 6 months; and patients with complete clinical and imaging data.
  Exclusion criteria: patients suffering from knee articular lesions for reasons other than KOA; patients with incomplete functions of essential organs, such as heart, liver, and kidney; pregnant or lactating women; and patients suffering from severe primary diseases, including cardiovascular, liver, and hemopoietic system diseases.

### 2.2. ROI Segmentation of Knee Joint on Account of Intelligent Algorithm

Before analyzing magnetic resonance images of the knee, segmentation of magnetic resonance images of different sequences was required. In this study, images of different sequences were segmented using a gray projection algorithm. The gray projection value obtained by summing the pixels in the whole image could reflect the gray-level information. Method of calculating pixels projected onto the horizontal axis is shown in the following equation:

(1) $$\begin{array}{c} G_{i}={\sum \text{pixel}\left(x,y\right)|}_{x=i}. \end{array}$$

In the above equation, *G*_*i*_ was the sum of the pixels projected onto *x*=*i*, and pixel(*x*, *y*) was the pixel value at coordinate (*x*, *y*). The projection to the vertical axis could be shown in the following equation:

(2) $$\begin{array}{c} G_{i}={\sum \text{pixel}\left(x,y\right)|}_{y=i}. \end{array}$$

In this study, the sharpness [12] was adopted to detect the ribbon area, which could be shown in the following equation:

(3) $$\begin{array}{c} S_{n}=\sum_{i=-55}^{55}\left|{\frac{G_{n}-G_{n+i}}{i}|}_{i\neq 0}\right|. \end{array}$$

ROI segmentation of knee joint mainly included longitudinal and transverse segmentation. In the process of vertical ROI segmentation, relaxation constants were introduced to segment it on account of relaxation principle [10]. The ROI boundary of knee joint on account of relaxation principle could be shown in the following equation:

(4) $$\begin{array}{c} \left\{\begin{array}{c} E_{t}=E_{j}-A,\\ E_{b}=E_{j}+B. \end{array}\right. \end{array}$$

In the above equation, *E*_*t*_ was the upper boundary of ROI of knee joint, *E*_*b*_ was the lower boundary of ROI of knee joint, and *E*_*j*_ indicated that the relaxation principle determined the upper and lower boundaries of ROI. *A* was upper boundary relaxation constant, and *B* was lower boundary relaxation constant.

The maximum entropy threshold method determined the binarization threshold by measuring the entropy of the gray histogram of the image. It had obvious advantages in background interference removal. If *P*(*x*) was the probability of occurrence of *x*, the information entropy could be shown in the following equation:

(5) $$\begin{array}{c} I\left(x\right)=-\int_{-\infty }^{\infty }P\left(x\right)\log\;P\left(x\right)\text{d}x. \end{array}$$

If the probability of occurrence of gray level *i* was *P*(*i*), the segmentation threshold was *T*, [0, *T*] was the background gray level value, and [*T*+1,255] was the foreground of pixel points, the probability of gray level in the background and foreground could be shown in the following equation:

(6) $$\begin{array}{c} \left\{\begin{array}{cc} P_{i}=\frac{P_{i}}{P_{T}}, & i\in \left[0,T\right],\\ P_{i}=\frac{P_{i}}{\left(1-P_{T}\right)}, & i\in \left[T+1,255\right]. \end{array}\right. \end{array}$$

In the above equation, *P*_*T*_ represented the proportion of pixels within the range of [0, *T*] to pixels in the whole image.

*D* stood for background and *E* stood for foreground, and the information entropy of background and foreground could be shown in the following equation:

(7) $$\begin{array}{c} \left\{\begin{array}{c} I_{D}=-\sum_{i=0}^{T}P\left(i\right)\log\;P\left(i\right),\\ I_{E}=-\sum_{i=T+1}^{255}P\left(i\right)\log\;P\left(i\right). \end{array}\right. \end{array}$$

Gray histogram reflected the occurrence frequency of each gray level in the image [13]. If the gray level is *i*, and *i* ∈ [0,255] and *N*(*i*) were the number of pixels in the image, the calculation method of *N*(*i*) could be shown in following equations:

(8) $$\begin{array}{c} N\left(i\right)=\sum_{x=0}^{W-1}\sum_{y=0}^{H-1}P\left[G\left(x,,y\right)\right], \end{array}$$

(9) $$\begin{array}{c} P\left[G\left(x,,y\right)\right]=\left\{\begin{array}{cc} 0, & G\left(x,y\right)\neq i,\\ 1, & G\left(x,y\right)=i. \end{array}\right. \end{array}$$

In the above equation, *G*(*x*, *y*) pixel point was gray value of (*x*, *y*). *W* was image width, and *H* was the height of the image.

**Figure 1:**
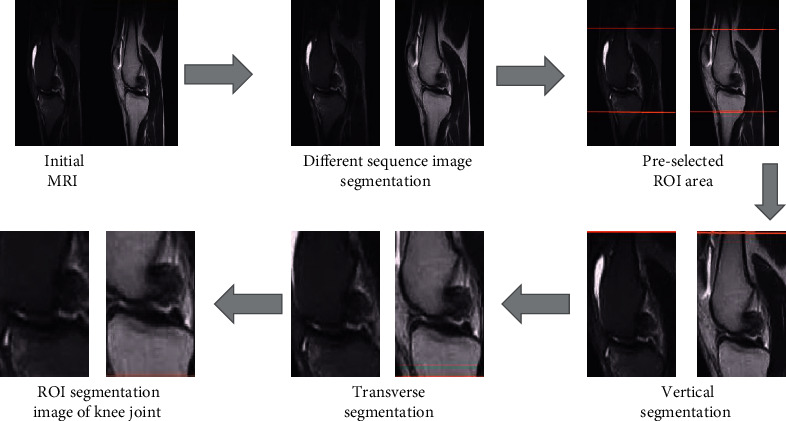
Specific flow chart of ROI segmentation of knee joint on account of intelligent algorithm.

Due to the gray projection algorithm of the initial magnetic resonance, images of different sequences were segmented. Relaxation constants were introduced to segment the knee preselected ROI region vertically and horizontally on account of the relaxation principle. Finally, ROI segmentation image was obtained. The specific process of knee ROI segmentation on account of intelligent algorithm in this study is shown in Figure 1.

### 2.3. Magnetic Resonance Knee Feature Point Extraction Based on Intelligent Algorithm

Most initial magnetic resonance images were exposed to unbalanced and uneven gray distribution phenomena [14]. If the gray level of X-ray image was set as 0∼255, it was the gray-level distribution range of initial magnetic resonance image and the gray-level distribution range of transformed magnetic resonance image, then the gray value of the image pixels transformed by the algorithm could be shown in the following equation:

(10) $$\begin{array}{c} H\left(x,y\right)=\frac{\left(j-k\right)\left[F\left(x,y\right)-g\right]}{f-g}. \end{array}$$

*F*(*x*, *y*) represented the gray value of pixel (*x*, *y*) before linear transformation, and *H*(*x*, *y*) was the gray value of pixel (*x*, *y*) after linear transformation.

In this study, Gaussian smoothing operator was used to denoise MRI, and one-dimensional Gaussian function could be shown in the following equation:

(11) $$\begin{array}{c} G\left(x\right)=\frac{1}{\sqrt{2\pi \sigma }}\exp\left(\frac{-x^{2}}{2\sigma^{2}}\right). \end{array}$$

The two-dimensional Gaussian function could be shown in the following equation:

(12) $$\begin{array}{c} G\left(\upsilon ,\mu \right)=\frac{1}{2\pi \sigma }\exp\left[\frac{-\left(\upsilon^{2}+\mu^{2}\right)}{2\sigma^{2}}\right]. \end{array}$$

The preprocessed image mainly realized edge detection according to the gray level of edge pixels. In this study, the algorithm was optimized on account of gradient operator to increase the precision of edge detection. If *F*(*x*, *y*) was the gray value of pixel (*x*, *y*), and the difference between the gray value of the pixel on the horizontal axis and the vertical axis was *dF*(*x*, *y*), the calculation method of gradient operator *H*_*x*_ and *H*_*y*_ could be shown in following equation:

(13) $$\begin{array}{c} \left[\begin{array}{c} H_{x}\\ H_{y} \end{array}\right]=\left[\begin{array}{c} \frac{dF\left(x,y\right)}{F\left(x,y\right)}\\ \frac{dF\left(x,y\right)}{F\left(x,y\right)} \end{array}\right]. \end{array}$$

Canny operator had the finest edge detection and good integrity, but it was difficult to select an appropriate threshold [15]. Active shape model (ASM) could represent contour shape through a vector and had significant advantages in image edge detection [16]. Therefore, this study extracted fine edge features on account of Canny edge detection results and ASM model. The shape vector of the image with *l* feature point was shown in the following equation:

(14) $$\begin{array}{c} K_{i}=\left(x_{i1},y_{i1},x_{i2},y_{i2},\cdots ,x_{il},y_{il}\right). \end{array}$$

(*x*_*il*_, *y*_*il*_) represented the coordinate of the *l* feature point in the *i*^th^ image, and then, *n* sample sets of shape vectors with length 2*l* were shown in the following equation:

(15) $$\begin{array}{c} J=\left(K_{1},K_{2},\ldots ,K_{n}\right). \end{array}$$

General alignment method and principal component analysis were used to eliminate the interference of nonshape information such as bone size and body position. Then, the edge contour was iterated on account of the local gray model. Then, the local texture of the *i*^th^ feature point was shown in the following equation:

(16) $$\begin{array}{c} G_{ij}={\left[G_{ij1},G_{ij2},\ldots G_{ij\left(2n+1\right)}\right]}^{T}. \end{array}$$

In the above equation, *G*_*ij*(2*n*+1)_ represented the gradient characteristic parameter of the 2*n*+1 adjacent point of the *i*^th^ feature point of the *j*^th^ image. Then, the mean and variance of *m* local textures was shown in following equations:

(17) $$\begin{array}{c} {\tilde{G}}_{i}=\frac{1}{m}\sum_{j=1}^{m}G_{ij}, \end{array}$$

(18) $$\begin{array}{c} S_{i}=\frac{1}{m}\sum_{j=1}^{m}\left(G_{ij}-{\tilde{G}}_{i}\right){\left(G_{ij}-{\tilde{G}}_{i}\right)}^{T}. \end{array}$$

Mahalanobis distance was shown in the following equation:

(19) $$\begin{array}{c} D_{s}=\left(G-{\tilde{G}}_{i}\right)S_{i-1}{\left(G-{\tilde{G}}_{i}\right)}^{T}. \end{array}$$

In the above equation, *G* was the new feature of feature point *i.* If the displacement generated by each feature point was arranged into vectors, it could be shown in the following equation:

(20) $$\begin{array}{c} M_{x}=\left(M_{x1},M_{x2},\ldots ,M_{xk}\right). \end{array}$$

ROI segmentation image was enhanced by linear grayscale transformation. Then, Gaussian operator was used to denoise the image and Gaussian smoothing was obtained. Canny operator was used to detect the edge of knee joint. Finally, the extraction map of knee joint feature points was obtained by local search strategy.

### 2.4. Preparation and Treatment of PRP

36 mL of peripheral venous blood was collected from patients, and 1/9 volume ratio sodium citrate injection was added for anticoagulation treatment. Red blood cells were extracted and centrifuged at 870 g for 15 min after centrifugation at 275 g for 10 min. The upper platelet-poor plasma was discarded. PRP was diluted with 0.9% normal saline, and then, 10% calcium chloride was added to activate platelets in PRP.

Patients were supine and partially disinfected. The point at which the lateral and upper edges of the patella met was selected as the entry point for puncture. All patients were injected with 2 mL PRP at (1500∼1800) × 10^9^/L. The knee joint of patients was passively flexion and extension for 3∼5 times after injection, and the puncture point was covered and wrapped with sterile dressing. The treatment was performed once a week for 3 consecutive weeks.

### 2.5. Test Methods and Evaluation Indicators of Knee Joint Magnetic Resonance

1.5 T magnetic resonance system was adopted. To be specific, the patients were instructed to take supine position. The examination was started after the knee was cut to 10° to 15°. During the examination, different sequences were applied according to different sections, such as PDWI-FS and T1-weighted image (T1WI) sequences in the coronal plane, PDWI-FS sequences in the sagittal plane, and T1WI and T2-weighted image (T2WI)-FS sequences in the transverse plane. The parameters were adjusted as follows: (1) PDWI-FS: time of echo (TE) of 34 ms and time of repetition (TR) of 3,800 ms; (2) T1WI: TE of 9.8 ms and TR of 500 ms; (3) T2WI-FS: TE of 85 ms and TR of 5,480 ms; and (4) interslice distance of 0.5 mm, matrix of 256 × 256, field of view of 18 cm, and slice thickness of 3 mm.

The distance of medial or external space of the knee joint, the ratio of medial or lateral space, and the amount of osteophyte of femoral or tibial bone, upper femoral angle, lower tibial angle, and tibial and femoral angle were obtained on the magnetic resonance films. These indicators were completed jointly by more than 1 attending orthopedic surgeon and radiologist. The calculation method of the ratio of medial and lateral space was shown in the following equation:

(21) $$\begin{array}{c} d=\frac{D_{i}}{D_{o}}. \end{array}$$

In the above equation, *D*_*i*_ and *D*_*o*_ were medial joint and lateral joint space, respectively.

The Western Ontario and McMaster Universities arthritis index (WOMAC) score was used to assess daily life difficulty before and after treatment. Knee osteoarthritis MRI osteoarthritis knee score (KOA MOAKS) was adopted to evaluate the degree of joint injury. Visual analogue scale (VAS) was used to assess the degree of pain in KOA patients and to compare the difference before and after treatment.

### 2.6. Statistical Methods

SPSS 19.0 statistical software was used for data processing. *T*-test was used to compare the differences. Spearman correlation coefficient was adopted to analyze the correlation between MOAKS score and WOMAC OA index. *P* < 0.05 indicated statistically significant difference.

## 3. Results

### 3.1. Analysis of Edge Detection Results of Knee Joint Magnetic Resonance Based on Intelligent Algorithm

**Figure 2:**
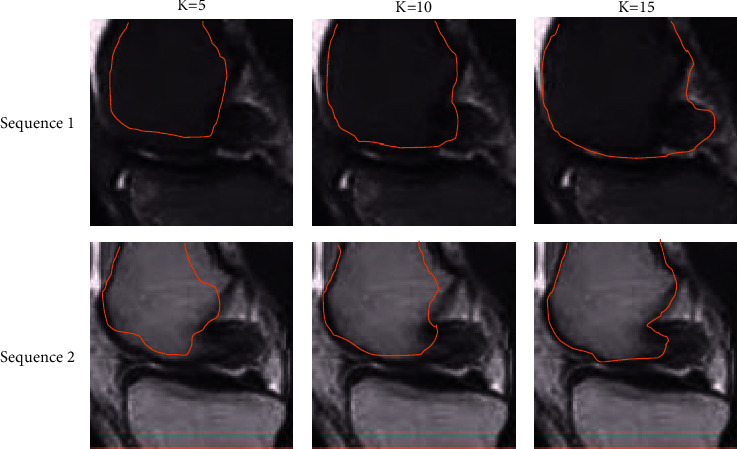
Results of MRI edge detection of knee joint based on intelligent algorithm.

The analysis of the edge detection results of knee joint magnetic resonance based on intelligent algorithm is given in Figure 2. With the increasing number of iterations *K*, the fit of the magnetic resonance contour extraction line was higher for the knee (marked in red) and the lower edge of the femur. It suggests that the accuracy of magnetic resonance knee edge detection becomes higher as the number of iterations increases.

### 3.2. Measurement and Analysis of Knee Joint Space Distance on Account of Intelligent Algorithm

**Figure 3:**
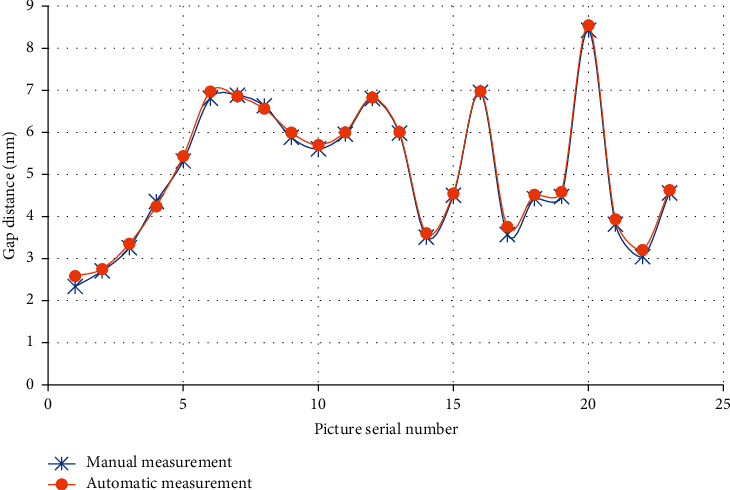
Measurement results of joint space by different methods.

Measurement and analysis of knee joint space distance on account of intelligent algorithm is shown in Figure 3. The results of automatic and manual detection of 23 knee joint space measurement had good consistency.

### 3.3. Analysis of Measurement Results of Knee Joint Correlation Angle on Account of Intelligent Algorithm

**Figure 4:**
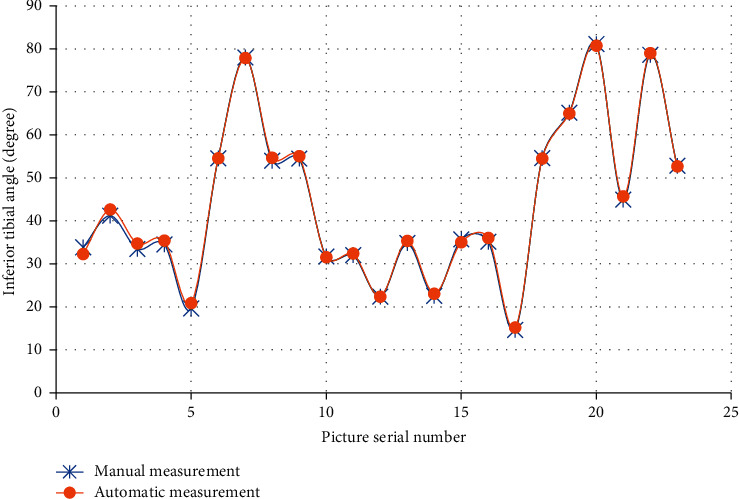
Comparison of inferior angle of femur measurement results with different methods.

**Figure 5:**
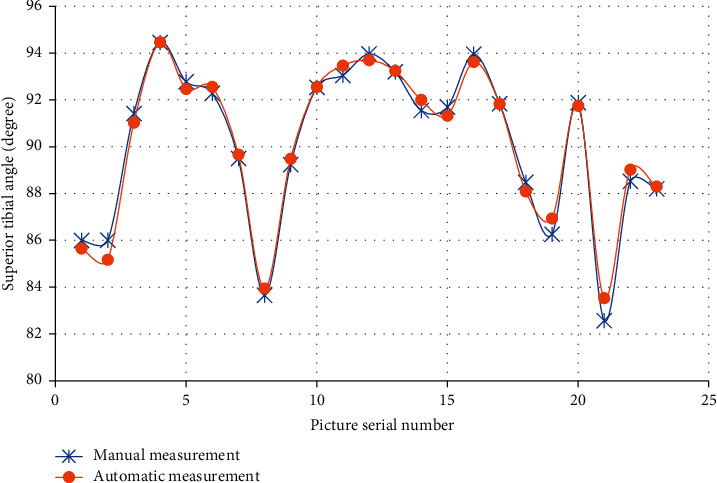
Comparison of superior angle of tibia measurement results with different methods.

**Figure 6:**
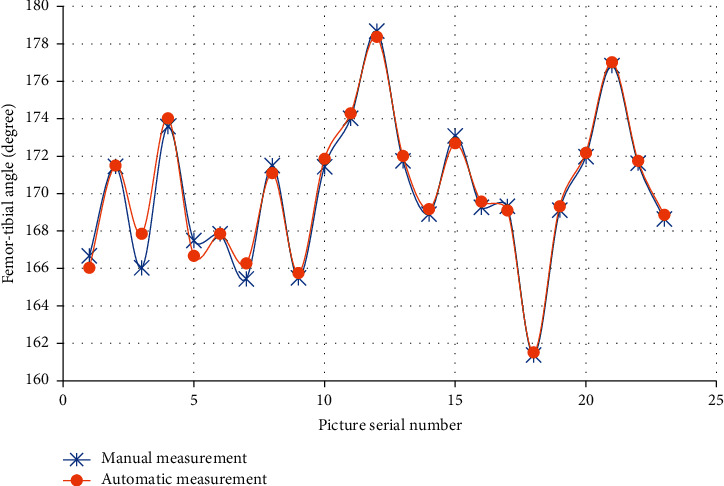
Comparison of TFA measurement results with different methods.

The analysis of detection results of inferior angle of femur, superior angle of tibia, and tibiofemoral angle (TFA) of the right leg in 23 images were analyzed, which are shown in Figures 4–6. The results of automatic detection and manual measurement of inferior angle of femur, superior angle of tibia, and TFA had good consistency.

### 3.4. Comparison of WOMAC Scores of KOA Patients before and after Treatment

**Figure 7:**
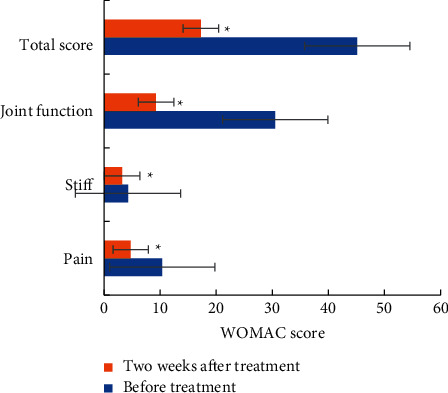
Comparison of WOMAC scores of KOA patients before and after treatment.  ^*∗*^indicated that the comparison with WOMAC scores before treatment showed statistical difference (*P* < 0.05).

Figure 7 shows the comparison of WOMAC scores of KOA patients before and after PRP treatment. After PRP treatment, the pain score, stiffness score, disability score, and WOMAC total score of patients were significantly decreased (*P* < 0.05), and the disability decrease was the highest.

**Figure 8:**
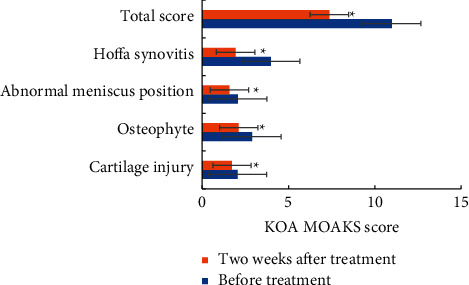
Comparison of KOA MOAKS scores of KOA patients before and after treatment.  ^*∗*^suggested that the comparison with KOA MOAKS scores before treatment demonstrated statistical differences (*P* < 0.05).

Figure 8 indicates the comparison of KOA MOAKS scores of KOA patients before and after treatment. After PRP treatment, the cartilage injury score, osteophyte score, meniscus malposition score, Hoffa synovitis score, and KOA MOAKS total score of patients were obviously decreased (*P* < 0.05). The greatest decrease occurred in Hoffa synovitis score.

### 3.5. Correlation Analysis between MOAKS Score and WOMAC OA Index

**Figure 9:**
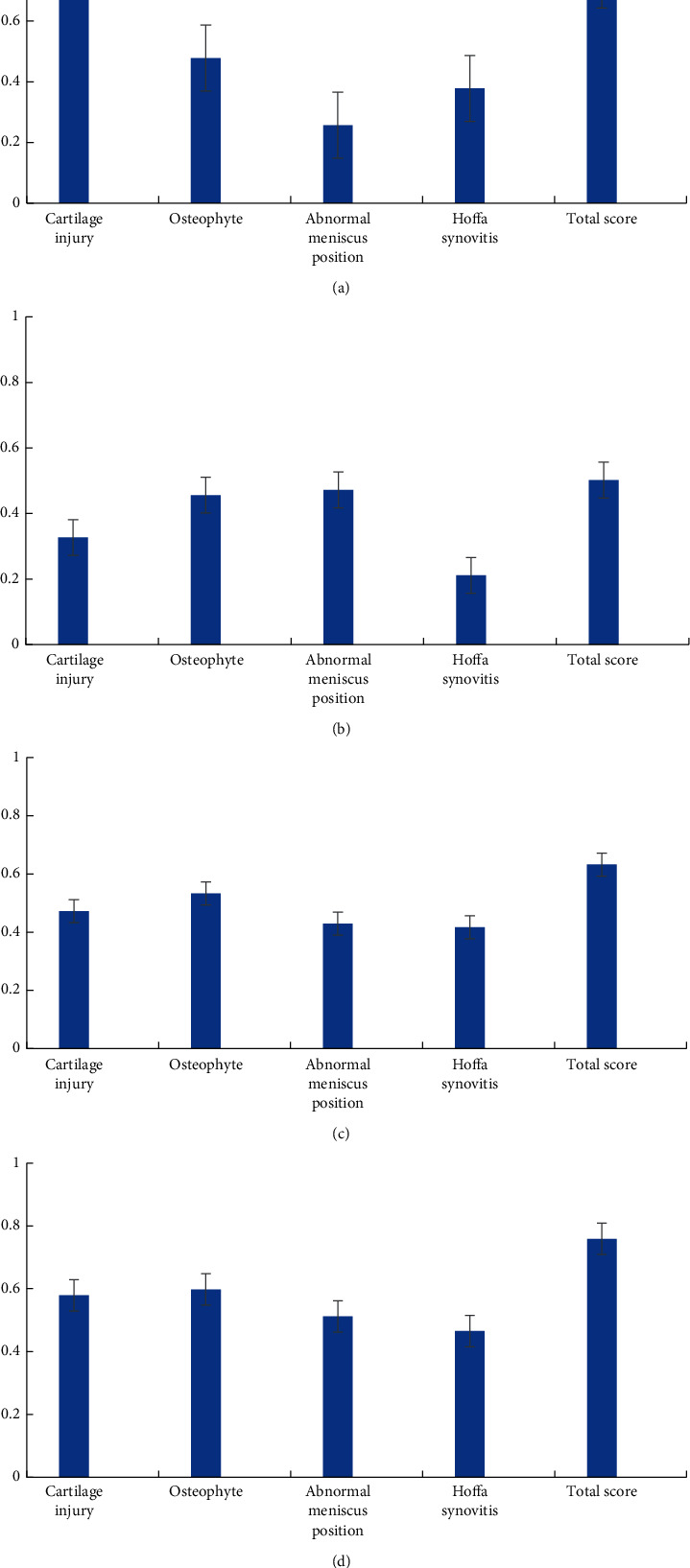
Correlation analysis between MOAKS score and WOMAC OA score for patients after PRP treatment. (a) The correlation between MOAKS score and pain score; (b) the correlation between MOAKS score and stiffness score; (c) the correlation between MOAKS score and disability score; (d) the correlation between MOAKS score and WOMAC OA total score.

Figure 9 reveals the correlation analysis of MOAKS score with pain score, stiffness score, disability score, and WOMAC OA total score after PRP treatment. MOAKS score has a good correlation with pain score, stiffness score, disability score, and WOMAC total score (*P* < 0.05) of which the correlation coefficient between bone marrow injury and pain is the highest (*r* = 0.825), and the correlation coefficient between MOAKS total score with pain and WOMAC total score is higher, 0.752 and 0.759, respectively.

### 3.6. Comparison of VAS Scores before and after Treatment

**Figure 10:**
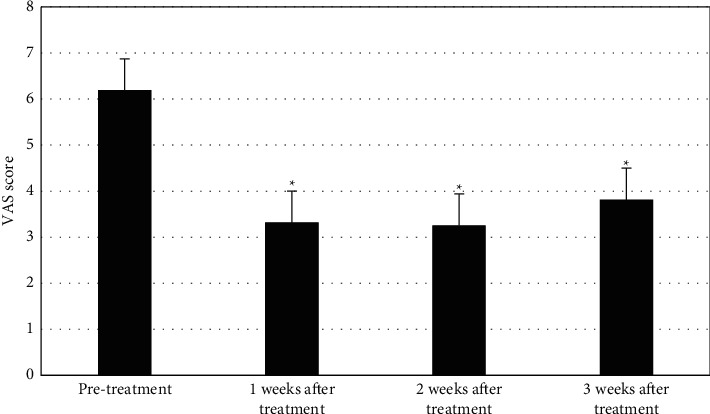
Comparison of VAS scores before and after treatment.  ^*∗*^represented statistical differences compared with those before treatment (*P* < 0.05).

The comparison and analysis of VAS scores of KOA patients before and after PRP treatment are shown in Figure 10. VAS scores of patients decreased first and then stabilized with the extension of time after treatment. VAS scores of patients at 1, 2, and 3 weeks after treatment were all lower than that before treatment (*P* < 0.05).

## 4. Discussion

KOA is a degenerative disease involving synovial membrane and knee joint. The main clinical manifestations are articular cartilage osteogenesis and cartilage destruction [10, 12]. KOA is common and frequent among elderly patients. The incidence of KOA among female patients is obviously higher than that among male patients [13, 14]. The current main therapeutic methods of Western medicine include oral drugs, local application, physiotherapy and rehabilitation, articular cavity injection, and knee joint replacement. The main therapeutic methods of Chinese medicine include treatment based on syndrome differentiation-based oral Chinese medicine decoction, plaster application, acupuncture, massage, and various special stitches. Pain is the most significant clinical manifestation among KOA patients. Relevant studies reveal that ozone combined with sodium hyaluronate treatment can be analgesic and anti-inflammatory. Besides, the therapy can obviously improve soft tissue functions, repair soft tissue damage, and show positive significance in accelerating the recovery of patients with KOA.

Among the examination methods for KOA, magnetic resonance examination is clearer than X-ray examination. It can judge and grade patients' signs, such as articular space by K-L classification. After treatment, the knee articular space becomes narrow [11, 15, 16]. Magnetic resonance shows good effects in the detection of knee articular space, inferior angle of femur, and superior angle of tibia [17, 18]. Intelligent algorithm-based automatic detection algorithm is very effective in the segmentation of MRI images. Among KOA patients, cartilage and osteophyte damage form at the joint edges [19]. Magnetic resonance can show different levels of narrow articular space, especially interior articular space [20]. Current clinical drug therapies are usually repeated so that it can cure KOA. The current study results reveal that PRP can promote the proliferation of osteoblast, the synthesis of cartilage and bone matrix, and the healing of soft tissues [21]. In addition, it was also found that PRP is anti-inflammatory in the treatment of KOA [22]. PRP can promote cartilage repair and shows potential utilization value in KOA treatment [23]. PRP is applied in orthopedics department more and more widely with various advantages. It is easy to obtain PRP, which does little harm to human body. Platelets with high concentrations are rich in a variety of growth factors and can effectively promote tissue healing [24].

Intelligent algorithm-based magnetic resonance segmentation algorithm was used to detect the clinical therapeutic effects of PRP on KOA. The difference between automatic magnetic resonance detection indexes of KOA patients and artificial measurement results was analyzed. Besides, KOA MOAKS and WOMAC before and after PRP treatment were compared. The research results showed that the detection results of knee articular space scores, inferior angle of femur, superior angle of tibia, and TFA by automatic magnetic resonance KOA diagnostic model were entirely consistent with artificial detection results. The results indicated that magnetic resonance played a positive role in the diagnosis of KOA and showed high clinical application values. Relevant studies suggested that the combined application of hyaluronic acid (HA) and PRP could repair degraded cartilage and delay the progression of KOA [22]. Raeissadat et al. [25] compared the short-term and long-term therapeutic effects of articular injection of HA, PRP, plasma rich in growth factor (PRGF), and ozone for patients with KOA. Consequently, they found out that the improvement of the symptoms of the patients only in PRP and PRGF groups lasted for 12 months, which showed long-term therapeutic effects. After the treatment, the total scores of knee lateral area, interior area, central area, and patellar area after treatment were all remarkably lower than those before the treatment (*P* < 0.05). After treatment, knee articular KOA MOAKS score and WOMAC were both lower than those before treatment (*P* < 0.05). MOAKS score and WOMAC score showed good correlation (*P* < 0.05). Barrow injury and pain-related coefficient were the highest, and the relevant coefficients of total MOAKS pain score and total WOMAC score were high. VAS score for patients after treatment was apparently lower than that before treatment (*P* < 0.05). The above results demonstrated that PRP could effectively improve the pain and articular pain among KOA patients and restore the functions of knee joint. PRP played a positive role in the treatment of KOA patients.

## 5. Conclusion

This study was on account of gray projection algorithm, adaptive binarization algorithm, ASM algorithm, and other intelligent algorithms to establish automatic diagnosis of MRI KOA. It aimed to discuss evaluation of MRI on account of intelligent algorithm on clinical effect of treatment of refractory pain in KOA with PRP. The results showed that the automatic diagnostic model of MRI KOA detection results on account of intelligent algorithm was highly consistent with those of manual detection. KOA patients were treated with PRP, and changes in knee KOA MOAKS scores and WOMAC scores were detected in magnetic resonance examinations before and after treatment. However, there were still some deficiencies in this study. In this study, the running time of automatic diagnostic model on account of intelligent algorithm and its specific detection accuracy were not further analyzed. In future experiments, the sample size will be further expanded to verify the running time, detection accuracy, sensitivity, and other parameters of the model established in [26] this study. In summary, automatic diagnostic model of MRI KOA on account of intelligent algorithm in this study could effectively evaluate the clinical efficacy of PRP in treating refractory pain in KOA. This provided a reference basis for the diagnosis and treatment of KOA patients.

## Data Availability

The data used to support the findings of this study are available from the corresponding author upon request.
